# ^18^F-FDG PET/CT Imaging In Oncology

**DOI:** 10.4103/0256-4947.75771

**Published:** 2011

**Authors:** Ahmad Almuhaideb, Nikolaos Papathanasiou, Jamshed Bomanji

**Affiliations:** Institute of Nuclear Medicine, University College London Hospitals National Health Service Trust, London, United Kingdom

## Abstract

Accurate diagnosis and staging are essential for the optimal management of cancer patients. Positron emission tomography with 2-deoxy-2-[fluorine-18]fluoro- D-glucose integrated with computed tomography (^18^F-FDG PET/CT) has emerged as a powerful imaging tool for the detection of various cancers. The combined acquisition of PET and CT has synergistic advantages over PET or CT alone and minimizes their individual limitations. It is a valuable tool for staging and restaging of some tumors and has an important role in the detection of recurrence in asymptomatic patients with rising tumor marker levels and patients with negative or equivocal findings on conventional imaging techniques. It also allows for monitoring response to therapy and permitting timely modification of therapeutic regimens. In about 27% of the patients, the course of managment is changed. This review provides guidance for oncologists/ radiotherapists and clinical and surgical specialists on the use of ^18^F-FDG PET/CT in oncology.

Cancer is one of the leading causes of death worldwide. Accurate diagnosis, staging and restaging are essential for the optimal therapeutic management of cancer patients. Positron emission tomography (PET) with 2-deoxy-2-[fluorine-18]fluoro-D-glucose (^18^F-FDG), an analogue of glucose, provides valuable functional information based on the increased glucose uptake and glycolysis of cancer cells and depicts metabolic abnormalities before morphological alterations occur. ^18^F-FDG PET/CT acquires PET and CT data in the same imaging session and allows accurate anatomical localization of the lesions detected on the ^18^F-FDG PET scan (**Figure 1**). Following its introduction, integrated PET/CT rapidly gained clinical acceptance, and in the last decade it has become an important imaging tool in routine clinical oncology (**Table 1**).

**Table 1:** Clinical indications of 18F-FDG PET/CT in oncology (include but are not limited to the following).

Evaluating the extent of disease in known malignancies (staging/restaging). Detecting tumor recurrence, in the presence of elevated tumor markers but no clinical or morphological evidence of disease. Searching for an unknown primary when metastatic disease is the first clinical presentation or when patients present with paraneoplastic syndrome. Differentiating benign from malignant lesions. Evaluating disease response to chemotherapy or radiotherapy. Selecting tumor region for biopsy guidance. Pre-surgical planning. Radiotherapy planning with therapeutic and palliative intent.

Modified from the European Association of Nuclear Medicine (EANM) and Society of Nuclear Medicine (SNM).

**Figure 1:**
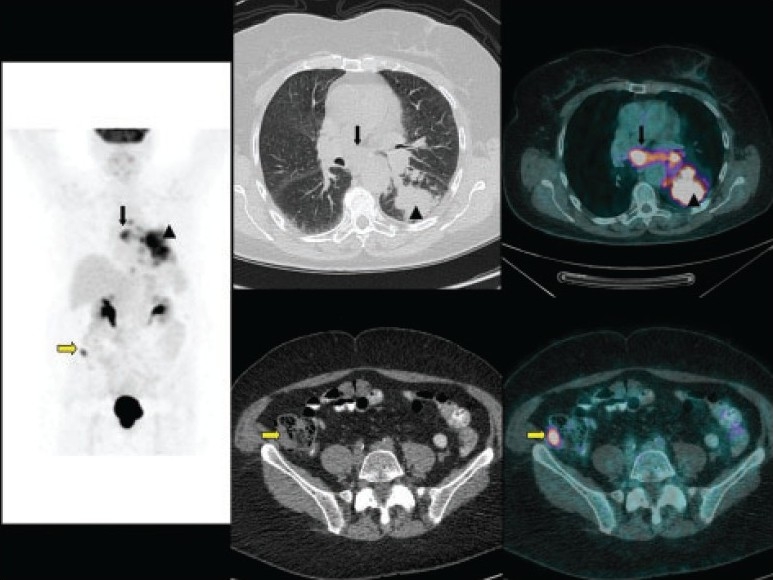
A 73-year-old woman who came for initial staging of non-small cell lung cancer. Maximum-intensity-projection (MIP) image (left panel), CT images (middle panels) and fused images (right panels) of 18F-FDG PET/CT show the primary tumor (arrow head) with mediastinal nodal metastases (black arrow). Incidental right iliac fossa small focal uptake (yellow arrow) is noted, which cross-correlated to a small soft tissue lesion in the cecum and turned out to be a synchronous primary adenocarcinoma.

^18^F-FDG PET/CT is more sensitive and specific in certain cancers and has been applied primarily as a staging and restaging tool that can guide patient care. It has also been used to distinguish responders from nonresponders before any reduction in tumor size occurs. In some tumors, e.g., lymphoma, non-small cell lung cancer and esophageal cancer, reduction in the ^18^F-FDG PET activity within days or weeks of initiating therapy correlates significantly with prolonged survival and other clinical endpoints now used in drug approvals.

There is evidence that ^18^F-FDG PET/CT is particularly useful for detecting recurrence, especially in asymptomatic patients with rising tumor marker levels and those with negative or equivocal conventional imaging findings. Yet there are some limitations and areas of uncertainty, mainly regarding the lack of specificity of ^18^F-FDG uptake and the variable avidity of some cancers for this tracer. This article reviews the main applications, advantages and limitations of ^18^F-FDG PET/CT in oncology.

## METHODS

A search was performed to identify mainly all published randomized controlled trials and systematic reviews in the English language literature. An additional search was performed to identify relevant unpublished systematic reviews. These publications comprised both retrospective and prospective studies of variable methodological quality. The consequences of false-positive and false-negative test results when evaluating the clinical usefulness of tests, as well as the impact of ^18^F-FDG PET/CT on the management of cancer patients, were also reviewed.

### Breast Cancer

^18^F-FDG PET/CT has no role in the diagnosis of primary breast cancer as its ability to detect small and/ or noninvasive carcinomas is poor, with an overall sensitivity of only 68% for tumors of size <2 cm.^1^,^2^ For axillary nodal staging, ^18^F-FDG PET/CT has variable sensitivity (79%-94%) and specificity (86%-92%),^3^,^4^ and therefore the predictive accuracy is insufficient to recommend this modality for routine use.^5^

**Figure 2:**
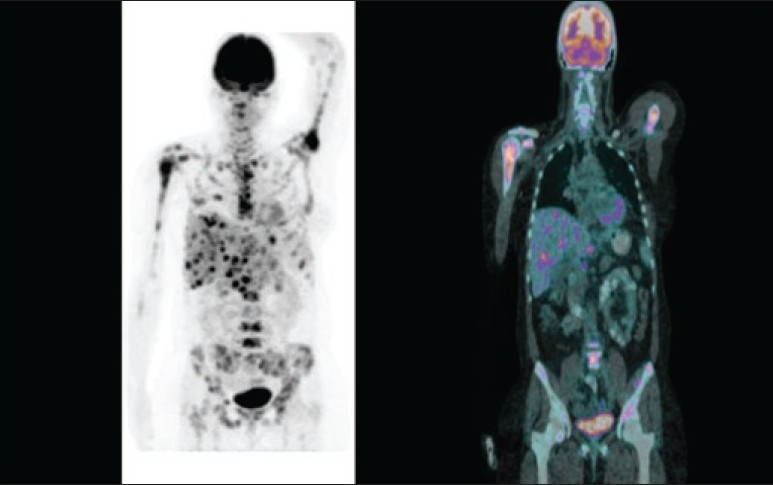
A 66-year-old woman who came for restaging of breast cancer. MIP image (left panel) and coronal fused images (right panel) of 18F-FDG PET/CT showed extensive hepatic and bony metastases.

The most important current clinical applications of ^18^F-FDG PET/CT in breast cancer patients are for the detection and evaluation of recurrent or metastatic disease (**Figure 2**) and for monitoring response to therapy.^6^ In a patient-based analysis, it was shown that ^18^F-FDG PET/CT has a high overall sensitivity, specificity and accuracy for the detection of locoregional recurrence (89%, 84% and 87%, respectively) and distant metastases (100%, 97% and 98%, respectively) (**Table 2**) and is also more sensitive than the serum tumor marker CA 15-3 in detecting relapsed disease.^7^.

**Table 2:** Sensitivity, specificity and accuracy of 18F-FDG PET/CT in different tumor types and settings.

	Sensitivity (%)	Specificity (%)	Accuracy (%)	References
**Breast cancer**				
Locoregional recurrence	89	84	87	7
Distant metastasis	100	97	98	7
Early response assessment	83-100	85-94	88-91	8
**Colorectal cancer**				
Recurrence	89	92	90	12
Intra-abdominal/extrahepatic recurrence	88	94	92	12
Extra-abdominal and/or hepatic recurrence	95	100	99	12
**Oesophageal cancer**				
Metastases (M-staging)	43-78	93-99	62-86	30-34
Recurrence (locoregional and distant)	94	82	87	42
**Head and neck cancer**				
Initial staging (nodal)	94	84	90	46, 47
Restaging/recurrence	88	78	86	55
**Lung cancer**				
Solitary pulmonary nodule	81-100	63-100		90-92
Mediastinal staging (N2/N3)	67-92	82-99	84-96	65-68
Recurrence	93-100	89-92		86-88
Response to treatment (≥80% threshold)	90	100	96	89
**Lymphoma**				
Initial staging and restaging (HL)	86	96		95
Nodal involvement in HL or high-grade NHL	94	100		96
Organ involvement in HL or high-grade NHL	88	100		96

Detection of a decrease in the standardized uptake value (SUV) to a level below 55% of the baseline study is a powerful tool in monitoring histopathological response to chemotherapy for locally advanced breast cancers. Using this criterion, ^18^F-FDG PET/CT was found to have a sensitivity of 100%, a specificity of 85% and an accuracy of 88% in identifying responders after the first cycle, while corresponding values after the second cycle were 83%, 94% and 91%.^8^ After a single pulse of chemotherapy, ^18^F-FDG PET was able to predict complete pathological response with a sensitivity of 90% and a specificity of 74%.^9^ The reported overall survival in ^18^F-FDG PET/CT nonresponders is 8.8 months, compared with 19.2 months in responders.^10^ In the case of bone metastases, the responding bony lesion may become more sclerotic on the CT component of ^18^F-FDG PET/CT while its ^18^F-FDG activity reduces, which is a sign of bone healing.

### Colorectal Cancer

In colorectal cancer, ^18^F-FDG PET/CT plays a pivotal role in the detection of recurrent disease, the assessment of residual post-therapy masses, the localization of recurrence in patients with an unexplained rise in serum carcinoembryonic antigen (CEA) and the staging of patients before surgical resection of local recurrence and distant metastatic disease.^11^ For the detection of intra-abdominal but extrahepatic colorectal recurrence, the sensitivity of ^18^F-FDG PET/CT is 88%; the specificity, 94%; and accuracy, 92%. For extra-abdominal and/ or hepatic recurrence, the sensitivity is 95%; specificity, 100%; and accuracy, 99%. The overall reported average sensitivity, specificity and accuracy for detecting recurrent disease are 89%, 92% and 90%, respectively (**Table 2**).^12^

**Figure 3:**
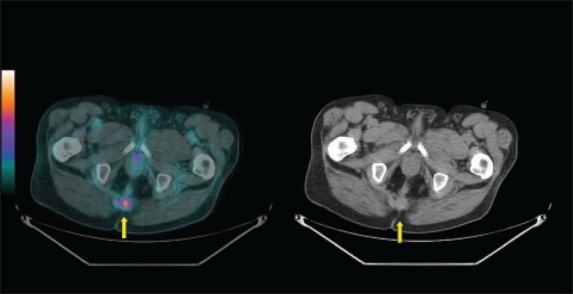
A 72-year-old man who had unexplained elevation of CEA during his follow-up after treatment of anorectal cancer. Fused (left panel) and CT (right panel) images of 18F-FDG PET/CT scan showed recurrent avid disease at the residual surgical bed soft tissue density (yellow arrow).

The residual pelvic soft tissue abnormalities frequently seen in the tumor bed region after therapy usually complicate the detection of local recurrence by the conventional imaging techniques.^13^,^14^ Abnormal ^18^F-FDG activity in a residual pelvic soft tissue lesion after 6 months from the completion of radiotherapy most likely represents tumor recurrence, and accuracy and positive predictive value (PPV) are even higher after 12 months.^11^,^15^,^16^ Elevated CEA levels are seen in two-thirds of patients with recurrent colorectal cancer.^17^–^20^ ^18^F-FDG PET/CT is recommended for patients with an unexplained increase in serum CEA level after primary curative treatment of colorectal cancer, since it changes the course of management in 59% to 68% of the patients (**Figure 3**).^11^,^21^–^23^

^18^F-FDG PET/CT is emerging as a potentially valuable technique in radiotherapy planning, as well as in the prediction and evaluation of response to therapy.^11^,^24^ The use of ^18^F-FDG PET/CT for preoperative radiotherapy planning in rectal cancer significantly alters both the gross tumor volume and the clinical target volume, with a mean increase in size of 25% and 4%, respectively.^25^

### Esophageal Cancer

**Figure 4:**
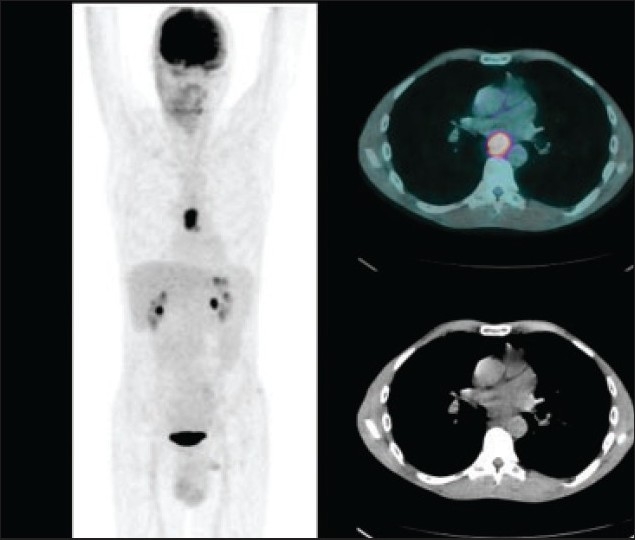
A 56-year-old man who came for initial staging of esophageal cancer. The MIP (left panel) and axial fused (right upper panel) and axial CT (right lower panel) images of 18F-FDG PET/CT showed the primary mid-esophageal tumor with no evidence of FDG-avid distant metastases.

Endoscopic ultrasound (EUS) provides more accurate and cost-effective T-staging and N-staging than ^18^F-FDG PET/CT and conventional CT^26^–^28^ and remains the standard for local tumor evaluation.^29^ The most important role of ^18^F-FDG PET/CT in the initial staging of esophageal cancer lies in M-staging (**Figure 4**) through its ability to identify unexpected metastases (i.e., metastases not visible on conventional imaging), which are present in up to 30% of the patients. ^18^F-FDG PET/CT has better sensitivity, specificity and accuracy (43%-78%, 93%-99% and 62%-86%, respectively) than CT and EUS for the detection of distant metastases (**Table 2**).^30^–^34^ In M-staging, the addition of ^18^F-FDG PET/CT results in up-staging of 15% to 20% and down-staging of 5% to 7% of the patients.^34^,^35^ In addition, synchronous primary tumors are identified in 5.5% of patients, of which 75% are not identified by conventional imaging.^36^

Assessment of tumor response to neoadjuvant therapy by ^18^F-FDG PET/CT has been found to be an important prognostic factor,^37^ with a reported diagnostic accuracy of 85%; this is similar to the diagnostic accuracy of EUS (86%) and significantly higher than that of conventional CT (54%).^38^

In patients with squamous cell carcinoma of the esophagus and some inoperable cases, ^18^F-FDG PET/CT plays an important role in radiotherapy planning,^39^–^41^ with a reported modification of gross tumor volume in 56% of the patients and alteration of the planning treatment volume in 53%.^41^

^18^F-FDG PET is a highly sensitive tool for the detection of regional and distant recurrences, with a reported sensitivity, specificity and accuracy of 94%, 82% and 87%, respectively, in comparison to 81%, 82% and 81% for conventional imaging. Furthermore, ^18^F-FDG PET depicted recurrences in 12% of the patients with negative or equivocal findings on conventional imaging.^42^

### Head and Neck Cancer

^18^F-FDG PET/CT has an impact on the assessment of both newly diagnosed and previously treated patients with head and neck cancer.^43^ ^18^F-FDG PET/CT alters the initial clinical staging and TNM category of the tumor in 14% to 57% of the patients when compared with CT-based evaluation alone,^44^,^45^ and has an accuracy of approximately 90% compared with 86% for conventional CT.^46^,^47^

The reported sensitivity and specificity of standard ^18^F-FDG PET/CT for the detection of lymph node metastases in a per-patient analysis were 94% and 84%, respectively (**Table 2**), in comparison to 78% and 84% for conventional CT.^48^

^18^F-FDG PET/CT has been found to identify synchronous primaries in 8.1%, distant metastases in 15.4% and the site of an unknown primary in 73% of the patients with head and neck cancer.^46^ In addition, it alters the initial management in 18% to 37% of the patients.^46^,^49^ The impact of ^18^F-FDG PET/CT on radiotherapy planning is especially important: planning is changed in 29% of the patients,^50^ with an alteration in the gross tumor volume in 57% of the patients.^45^ It has been reported that the gross tumor volume is statistically significantly larger with 18F-FDG PET/CT–based assessment than with CT-based assessment.^51^,^52^ There is still a high risk of locoregional recurrence (18%-31%) and distant metastasis (20%-25%) despite aggressive treatment.^53^,^54^ The sensitivity, specificity and accuracy of ^18^F-FDG PET/CT in restaging patients with head and neck cancer are 88%, 78% and 86%, respectively.^55^

Postoperative, but pre-radiotherapy, ^18^F-FDG PET/CT evaluation within a median of 4 weeks after surgery has been found to alter the course of management in 15% of the patients.^56^ In addition, it has a higher accuracy than conventional CT when used at 4 to 8 weeks following the end of chemoradiotherapy, with an even higher sensitivity and specificity after 8 weeks.^57^

### Lung Cancer

Correct initial staging of non-small cell lung cancer (NSCLC) is important in distinguishing operable patients from those who are inoperable, but can benefit from neoadjuvant treatment.^58^ The American College of Chest Physicians guidelines^59^ recommend ^18^F-FDG PET for noninvasive staging owing to the low sensitivity and specificity of the commonly used conventional CT in mediastinal nodal staging. ^18^F-FDG PET/CT is a more accurate method and is the emerging standard test for preoperative diagnosis and staging of NSCLC; it changes the course of management in up to 52% of cases and has a major role in reducing the number of futile thoracotomies.^60^–^63^

Diagnostic accuracy and sensitivity of ^18^F-FDG PET/CT staging of lung cancer in terms of operability have recently been reported to be 79% and 64%, respectively, in comparison to 60% and 32% for conventional staging^64^ The initial reported sensitivity and specificity for ^18^F-FDG PET in mediastinal nodal assessment are 67% to 92% and 82% to 99%, respectively (Table 2), in comparison to 25% to 71% and 66% to 98% for CT alone. Overall, the correct stage is assessed by ^18^F-FDG PET in 85% to 96% of the cases as compared with 58% to 59% by conventional CT alone, and ^18^F-FDG PET has a negative predictive value (NPV) of 97% (CT, 87%).^65^–^67^ Sensitivity, specificity and accuracy of ^18^F-FDG PET/CT for the depiction of malignant nodes are 85%, 84% and 84%, respectively, in comparison to 70%, 69% and 69% for CT alone.^68^

**Figure 5:**
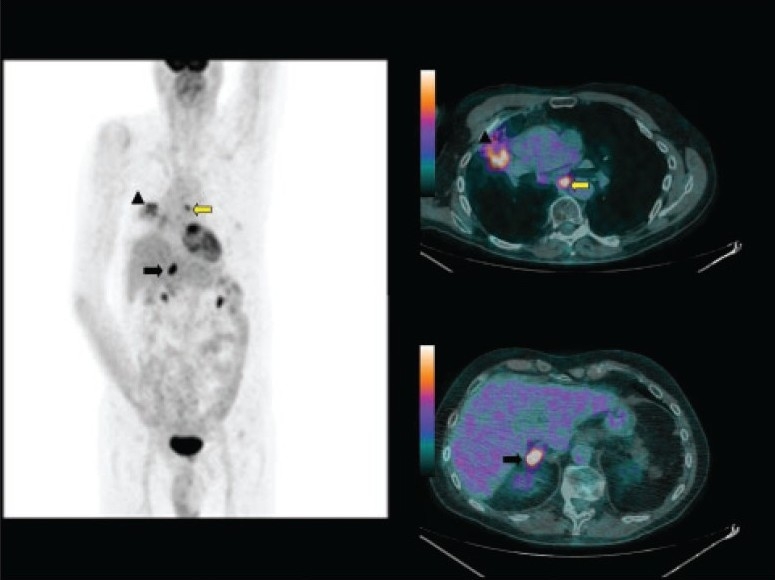
A 68-year-old man who came for initial staging of non-small cell lung cancer. MIP image (left panel) and fused images (right panel) of 18F-FDG PET/CT showed the primary tumor (arrow head) with mediastinal nodal involvement (yellow arrow) and extra-thoracic right adrenal metastasis (black arrow).

The high NPV of ^18^F-FDG PET/CT (up to 97%) for mediastinal disease^69^–^71^ has led to the recommendation to omit mediastinoscopy in patients with negative mediastinal ^18^F-FDG PET/CT.^70^,^72^,^73^ However, special attention should be paid to central tumors, which have a high incidence of occult N2 disease.^74^ If ^18^F-FDG PET/CT is positive, then mediastinoscopy is necessary to exclude a false-positive result.^59^ ^18^F-FDG PET/CT detects unexpected extrathoracic metastases (**Figure 5**) in 11% to 15% of asymptomatic patients, avoiding futile surgical intervention.^70^,^75^,^76^

^18^F-FDG PET/CT is useful for radiation therapy planning since it provides more accurate initial staging, allowing omission of elective radiation of clinically uninvolved nodal stations.^77^ In addition, its CT data may be used for radiation therapy planning if properly acquired.^78^ This modality can be successfully applied to patients with limited-disease small cell lung cancer for whom the treatment is concurrent chemoradiotherapy, with a reported 24% change in the treatment field.^79^

Post-treatment fibrosis and scarring are common, and ^18^F-FDG PET/CT is more accurate than conventional CT in detecting residual and recurrent disease, which allows more reliable treatment planning decisions.^80^–^83^ In addition, conventional CT alone has been shown to be suboptimal in mediastinal restaging after treatment.^84^,^85^ ^18^F-FDG PET has sensitivity of 93% to 100% and a specificity of 89% to 92% for detecting recurrent NSCLC.^86^–^88^ Patients with residual ^18^F-FDG uptake after treatment have a poor prognosis when compared to those without residual ^18^F-FDG uptake, taking into consideration the expected post-therapeutic inflammatory changes to avoid false-positive interpretation.^84^

Reduction in the baseline maximum SUV on ^18^F-FDG PET is predictive of a complete pathologic response with a sensitivity of 90%, a specificity of 100% and an accuracy of 96%, irrespective of the cell type or neoadjuvant treatment.^89^ Indeterminate solitary pulmonary nodules (SPNs) remain a clinical dilemma. ^18^F-FDG PET/CT currently should be reserved for cases where CT-guided fine-needle biopsy either is technically difficult or has been non-diagnostic.^80^ Compared with CT scan, ^18^F-FDG PET has similar sensitivity but better specificity in depicting malignancy in SPNs, the reported values ranging from 81% to 100% and from 63% to 100%, respectively.^90^–^92^

### Lymphoma

^18^F-FDG PET/CT is now an established standard in the initial staging, monitoring of response to therapy and restaging after treatment of patients with Hodgkin lymphoma (HL) and high-grade non-Hodgkin lymphoma (NHL).^93^ The clinical utility of ^18^F-FDG PET/CT depends on the pathological subtype but not necessarily on the tumor grade.^94^ ^18^F-FDG PET/CT shows a sensitivity of 86% and a specificity of 96%, in comparison to 81% and 41% with conventional CT alone, in disease assessment (presence or absence) of HL during both initial staging and restaging.^95^ In patients with HL or high-grade NHL, the sensitivity and specificity of ^18^F-FDG PET/CT for lymph node involvement are 94% and 100%, respectively, while for organ involvement they are 88% and 100% (**Table 2**).^96^

False-negative scans are noted in MALT (mucosal-associated lymphoid tissue) lymphomas, which are not highly metabolically active.^97^ Aggressive (high-grade) NHL typically shows more intense ^18^F-FDG activity in comparison to lower-grade NHL, although there is significant overlap between them.^96^ Detection of an FDG-avid lesion in a documented low-grade NHL should raise the suspicion of transformation to a higher-grade lymphoma.^97^,^98^ Infectious and/or inflammatory diseases are known causes of false-positive ^18^F-FDG PET/CT scans, and the possibility of their presence should be entertained at interpretation.^99^–^102^

**Figure 6:**
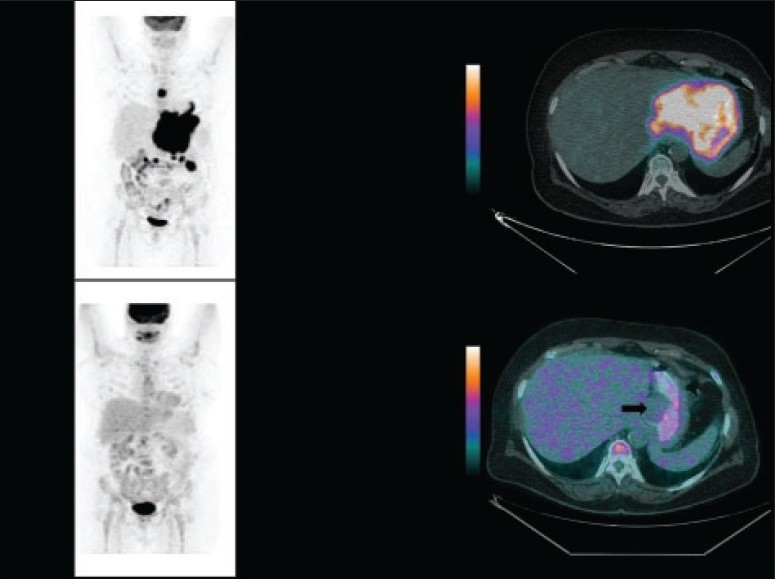
A 66-year-old woman diagnosed with Hodgkin lymphoma. The 18F-FDG PET/CT study (left and right upper panels) for initial staging showed nodal involvement above and below the diaphragm. 18F-FDG PET/CT after four cycles of chemotherapy (left and right lower panels) showed complete metabolic resolution of the disease with small non–FDG-avid residual soft tissue (black arrow on the fused image).

Residual post-therapy masses are seen in up to 85% of the cases of HL and up to 40% of the cases of NHL.^103^,^104^ Early interim ^18^F-FDG PET/CT results (after two to four cycles) correlate well with event-free survival in HL (**Figure 6**)^105^–^107^ and high-grade NHL.^108^,^109^ In high-grade NHL, the event-free survival at 2 years and 5 years has been reported to be 82% and 88.8%, respectively, for negative interim PET patients in compassion to 43% and 16.2%, respectively, for positive interim PET patients.^108^,^109^ In another study, the 2-year event-free survival in HL patients with negative interim ^18^F-FDG PET was 95% in comparison to 12.8% in those with positive interim ^18^F-FDG PET.^107^

### Thyroid Cancer

More than 90% of thyroid cancers are differentiated, comprising papillary and follicular carcinoma.^110^ In de-differentiated thyroid cancer, recurrent or metastatic tumor cells may lose the expression of sodium iodide symporter and have a decreased ability to concentrate radioiodine.^111^ A multicenter trial showed that the sensitivity of ^18^F-FDG PET is 85% in patients with raised thyroglobulin and negative 131I whole-body scans.^112^ In this subgroup of patients, ^18^F-FDG PET/CT alters clinical management in 23% to 51% of the pateints.^113^–^118^

### Urological Cancer

#### Renal cell carcinoma

^18^F-FDG PET/CT has limited sensitivity in the evaluation of metastatic Renal cell carcinoma (RCC), particularly for small metastatic lesions. However, a positive ^18^F-FDG PET/CT scan should be considered strongly suspicious for local recurrence or metastasis because of the high specificity and PPV of this test. A combined test (PET/contrast-enhanced CT) may be necessary if important management decisions are to be based on the test result.^119^

#### Prostate cancer

Currently there is no established role for ^18^F-FDG PET/CT in the assessment of prostatic cancer, since it has a low accuracy owing to the relatively low metabolic rate of the tumor as well as the interfering adjacent urinary excretion of the tracer. However, other new PET radiotracers such as 11C-choline and ^18^F-fluorocholine have shown promising results in the management of prostate cancer.^119^

#### Bladder cancer

Currently there is no established role for ^18^F-FDG PET/CT in the assessment of bladder cancer, since the high adjacent physiological urinary excretion of the tracer renders the signal-to-noise ratio unfavorable for lesion detection.

### Gynecological Cancers

#### Cervical cancer

^18^F-FDG PET(/CT) has a major role in preoperative staging of advanced cervical cancer and restaging after treatment.^120^,^121^ ^18^F-FDG PET has a sensitivity of 86%, a specificity of 94% and an accuracy of 92% for detection of para-aortic nodal metastases in patients with advanced cervical cancer and negative abdominal CT.^122^ Furthermore, preoperative evaluation with ^18^F-FDG PET influences patient management in 18% of patients; while in the case of recurrent cervical cancer, ^18^F-FDG PET shows an overall sensitivity of 86% to 94% and specificity of 76% to 100%.^123^ The 2-year progression-free survival rate is 86% for patients with a negative post-treatment scan in comparison to 40% for those with persistent abnormal ^18^F-FDG uptake.^124^

#### Ovarian cancer

^18^F-FDG PET(/CT) has a major role in the evaluation of recurrent ovarian cancer when there is an increase in serum CA-125 and inconclusive or negative conventional (CT/MRI) imaging.^121^ The reported sensitivity and PPV of ^18^F-FDG PET/CT for detection of recurrent disease at least 1 cm in size are 83.3% and 93.8%, respectively.^125^

### Cutaneous Melanoma

There is no role for ^18^F-FDG PET/CT in early cutaneous melanoma (American Joint Committee on Cancer stages I and II).^126^,^127^ In advanced (AJCC stages III and IV) and recurrent cutaneous melanoma, ^18^F-FDG PET shows 100% sensitivity for visceral and abdominal nodal metastases and 100% accuracy for superficial lymph node metastases, but lower sensitivity for pulmonary metastases.^128^ However, the CT component of a combined PET/CT scan would allow better evaluation of pulmonary metastases. The reported rate of synchronous tumor on ^18^F-FDG PET was 4.3%.^129^ ^18^F-FDG PET results in changes in staging in 12% to 34% of the patients^130^,^131^ and changes in overall management in 8% to 61% of the patients.^132^,^133^

### Brain Tumors

Sensitivity and specificity of ^18^F-FDG PET/CT in evaluating low-grade and recurrent tumors and treatment-induced changes are relatively low, mainly owing to the adjacent high physiological brain ^18^F-FDG activity; however, this can be improved significantly by co-registration with magnetic resonance imaging and potentially by delayed imaging. ^18^F-FDG PET/CT is capable of identifying anaplastic transformation of a documented low-grade tumor and has a prognostic value.^134^

### Pitfalls

**Figure 7:**
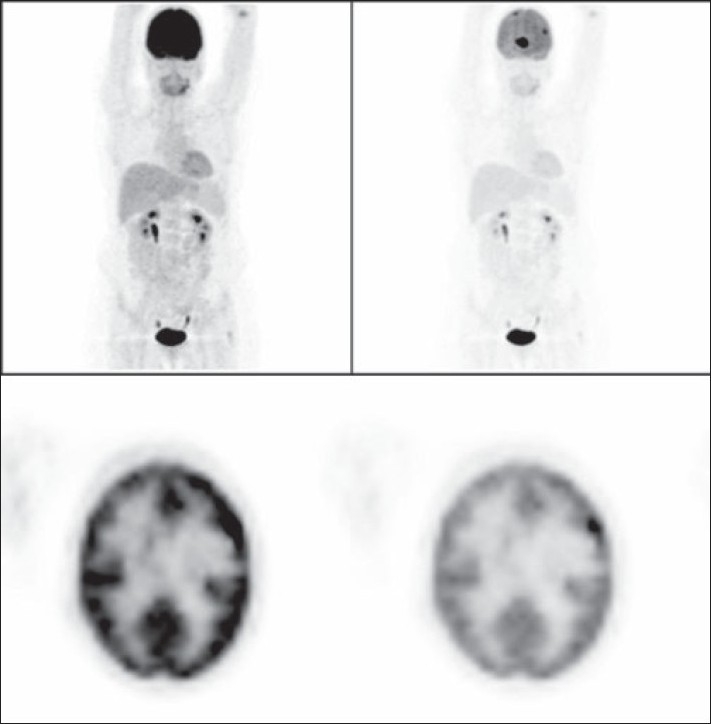
A 62-year-old woman with history of breast cancer. The left column images (MIP and axial PET images) show the normal-intensity images, which could hide metastatic deposits and give a false-negative result due to the physiological high background intensity of the brain. The same images after reducing their intensity on the right column show the metastatic deposits.

It is extremely important to consider some pitfalls of ^18^F-FDG PET/CT imaging during scan interpretation. The ability to detect tumors depends on various factors, such as their size, metabolic activity, the surrounding background activity and the serum glucose level. False-negative results may be obtained in small lesions (<7 mm), in tumors with a low metabolic rate (e.g., differentiated neuroendocrine tumors, prostate cancer, hepatocellular carcinoma, MALT and mucinous adenocarcinoma), in the presence of interfering cytostatic treatments that may decrease the tumor ^18^F-FDG uptake and when there is suboptimal preparation of patients with glucose intolerance or diabetes (since elevated serum glucose levels result in decreased FDG uptake in tumors owing to competitive inhibition). In addition, local high physiological FDG activity (as in the brain and the genitourinary tract) can render the signal-to-noise ratio unfavorable for lesion detection (**Figure 7**), and may give rise to a false-negative result by masking a malignant lesion.

On the other hand, activated macrophages, neutrophils, fibroblasts and granulation tissue show increased ^18^F-FDG activity; therefore, infectious/ inflammatory processes (e.g., granulomatous diseases, abscesses, active thyroiditis), post-surgical changes (healing surgical wounds, scars, stoma, tube placement) and post-radiation changes (active fibrosis, radiation pneumonitis) may demonstrate increased ^18^F-FDG activity and cause a false-positive result.

### The Future

As to the evolving role of ^18^F-FDG PET/CT and possible future directions for PET/CT, the need to evaluate early response to therapy remains, and there are no good imaging tools at present. Data shows that ^18^F-FDG PET/CT predicts not only response to therapy, but also further hard endpoints, such as time to progression. It is likely that more well-designed and large clinical studies on ^18^F-FDG PET/CT will expand its approved clinical indications in this context. Currently the majority of PET/CT investigations in oncology use ^18^F-FDG (glucose metabolic marker) as a tracer. However, the changing demand to evaluate tumor angiogenesis, tumor hypoxia, tumor cell proliferation and tumor receptors, has led to the development of other specific tracers, which will get greater clinical acceptance with time.
